# Affective states influence emotion perception: evidence for emotional egocentricity

**DOI:** 10.1007/s00426-020-01314-3

**Published:** 2020-03-23

**Authors:** Irene Trilla, Anne Weigand, Isabel Dziobek

**Affiliations:** 1 Berlin School of Mind and Brain, Humboldt-Universität zu Berlin, Unter den Linden 6, 10099 Berlin, Germany; 2 Department of Psychology, Humboldt-Universität zu Berlin, Unter den Linden 6, 10099 Berlin, Germany

## Abstract

Research in social cognition has shown that our own emotional experiences are an important source of information to understand what other people are feeling. The current study investigated whether individuals project their own affective states when reading other’s emotional expressions. We used brief autobiographical recall and audiovisual stimuli to induce happy, neutral and sad transient states. After each emotion induction, participants made emotion judgments about ambiguous faces displaying a mixture of happiness and sadness. Using an adaptive psychophysics procedure, we estimated the tendency to perceive the faces as happy under each of the induced affective states. Results demonstrate the occurrence of egocentric projections, such that faces were more likely judged as happy when participants reported being happy as compared to when they were sad. Moreover, the degree of emotional egocentricity was associated with individual differences in perspective-taking, with smaller biases being observed in individuals with higher disposition to take the perspective of others. Our findings extend previous literature on emotional egocentricity by showing that self-projection occurs when we make emotion attributions based on the other’s emotional expressions, and supports the notion that perspective-taking tendencies play a role in the ability to understand the other’s affective states.

## Introduction

The ability to understand the emotions of others is crucial to successfully function in social interactions. Due to a lack of direct access to other people's mind, we have to rely on indirect sources of information to infer how others feel. For example, we could use information about the context the other person is exposed to, or read their emotional expressions. While these external cues about the other can be valuable means to emotion understanding, research in social cognition has shown that one’s own affective state is also used to guide judgments about others’ emotional experiences (Silani, Lamm, Ruff, & Singer, 2013; Steinbeis and Singer, 2014). That is, people tend to project their own emotions when inferring what other people feel, a process known as emotional egocentricity. Self-projection can be an efficient heuristic, especially when our experiences are similar to the others. However, it can also lead to inaccurate emotion attributions unless egocentric inferences are adjusted to account for dissimilarities between oneself and the other person (Mitchell, 2009).

Emotional egocentricity has been studied using tasks in which participants are asked to make emotion judgments about themselves and about another person while being simultaneously exposed to either affectively congruent stimulation (e.g., both were touched by a pleasant material) or affectively incongruent stimulation (e.g., the participant received pleasant touch, while the target was touched by an unpleasant material; Silani et al., 2013). A tendency to project the own emotions onto others is typically indicated by emotion judgments that are biased towards the participants’ own affective states, particularly in incongruent conditions. Egocentric biases have consistently been observed in similar perspective-taking paradigms that used monetary reward and punishment (Steinbeis and Singer, 2014), as well as visuogustatory (Hoffmann, Singer, & Steinbeis, 2015) and audiovisual (von Mohr, Finotti, Ambroziak, & Tsakiris, 2019) stimulation, to induce congruent and incongruent affective states to the participant and the target.

A common feature of the existing emotional egocentricity tasks is that participants are not able to see the target's reactions to the affective stimulations, so emotion judgments are exclusively based on information about the type of stimulation the other is exposed to. Under these conditions, social cognitive processes such as perspective-taking may be activated to infer the other’s emotion. In particular, performance in these paradigms has been taken as an indicator of the participants’ self-other distinction abilities, as egocentric biases in this context are thought to reflect a failure to distinguish the representation of one’s own affective states from that of the other (Silani et al., 2013; Hoffmann, Koehne, Steinbeis, Dziobek, & Singer, 2016; Tomova, von Dawans, Heinrichs, Silani, & Lamm, 2014).

In daily life situations, however, we can often rely on more basic abilities that do not require perspective-taking processes to understand what others are feeling, such as emotion perception. Imagine, for example, that you give a present to a friend. You will probably first judge whether they liked it or not based on interpreting their emotional reactions when they unwrap it. Indeed, the accurate reading of emotional signals such as facial expressions (Lindner and Rosén, 2006), body postures (de Gelder, de Borst, & Watson, 2015) or speech prosody (Golan, Baron-Cohen, Hill, & Rutherford, 2007) has been shown to be key for understanding the affective states of others. Until now, however, little attention has been placed in studying egocentricity during perception-based emotion attribution.

Previous research on the influence of mood on emotion perception provide a first indication of egocentric biases when reading other’s emotional states. Studies inducing positive and negative affective states to participants have shown that emotional facial expressions are more easily recognized when they are congruent with the participant’s induced mood (Lee, Ng, Tang, & Chan, 2008; Niedenthal, Halberstadt, Margolin, & Innes-Ker, 2000; Qiao-Tasserit, Quesada, Antico, Bavelier, Vuilleumier, & Pichon, 2017; Schmid and Schmid Mast, 2010). These mood-congruency effects have been often contextualized under general cognitive theories of affect congruence, according to which affective states activate linked memory representations and facilitate the encoding and processing of affectively-congruent information (Forgas, 2017). However, mood-congruency effects could also reflect emotional egocentricity: they may be the result of an over-attribution of the own affective states to others. In line with this interpretation, biases in emotion perception seem to be stronger when the emotion experienced by the participant and the emotion expressed by the target are incongruent (Schmid and Schmid Mast, 2010). Compared to classic emotional egocentricity paradigms, however, these biases may stem from more implicit and unconscious processes of self-projection, rather than reflecting self-other distinction abilities.

The current study sought to revisit mood-congruent biases in emotion perception as a measure of emotional egocentricity. First, we developed a novel approach to estimate the degree to which the own affective states bias judgments of emotional facial expressions. Using a combination of brief autobiographical recall and audiovisual stimuli, we induced happy, neutral and sad transient states to the participants. After each emotion induction, participants completed a short emotion perception task in which they made binary decisions (“happy” or “sad”?) about the expression of faces displaying a mixture of happiness and sadness. We hypothesized that emotional judgments would be biased by the participants’ affective states, such that they would more likely judge the ambiguous faces as happy when feeling happy than when being sad.

Second, we predicted that the magnitude of egocentric biases during the emotion judgments would be related to the participants’ disposition to consider and react to other people’s experiences. In particular, we examined associations with two components of dispositional empathy measured with the Interpersonal Reactivity Index (IRI; Davis, 1980). On the one hand, the empathic concern scale taps into affective empathy and measures the tendency to react with feelings of sympathy and concern for unfortunate others (Davis, 1980). On the other hand, the perspective-taking scale assesses the tendency to adopt the point of view of another person, a facet of cognitive empathy (Davis, 1980). An association between mood-congruent biases and dispositional empathy would be an indication that the mood effects on emotion perception are related to processes of social cognition. Finally, we explored associations with autistic traits, as stronger egocentricity during cognitive mentalizing (Bradford, Hukker, Smith, & Ferguson, 2018; Pearson, Ropar, & de Hamilton, 2013) and deficits in emotion recognition (Uljarevic and Hamilton, 2013) are commonly observed in autism spectrum conditions (ASC).

## Methods

### Participants

Fifty German-speaking adults (31 females, *M*_age_ = 27.82, SD_age_ = 6.66, range_age_ = 19–44) were recruited for this study. An a priori power analyses using G*Power 3 (Faul, Erdfelder, Lang, & Buchner, 2007) estimated a sample of 49 participants (*α* = 0.05, power = 0.80, three measurement levels, within-subject repeated-measures analysis of variance) for a *η*_p_^2^ = 0.17. The effect size was determined based on the emotional egocentricity effects reported in Silani et al. (2013; behavioral experiment 1: *η*_p_^2^ = 0.074; behavioral experiment 2: *η*_p_^2^ = 0.277) and Hoffmann, Banzhaf, Kanske, Gärtner, Bermpohl, & Singer (2016; *η*_p_^2^ = 0.180).

Exclusion criteria included current psychiatric or neurological disorders, cognitive or neurological impairments, and psychoactive medication. All participants gave written informed consent and were financially remunerated for their participation. The study was conducted in compliance with the latest revision of the Code of Ethics of the World Medical Association (Declaration of Helsinki), and was approved by the Ethics Committee of the Psychology department at Humboldt-Universität zu Berlin.

### Materials and procedure

#### Emotional egocentricity paradigm

The emotional egocentricity paradigm comprised three blocks, corresponding to the three affective state manipulations applied to each participant (happy, neutral and sad). Each block was divided in two parts: an emotion induction, followed by an emotion perception task. MATLAB R2015b (The MathWorks, Inc., Natick, Massachusetts, United States) and the Psychophysics Toolbox extension (Brainard,^1997^; Kleiner, Brainard, Pelli, Ingling, Murray, & Broussard,^ 2007^) were used for stimulus presentation.

##### Emotion induction

A combination of a brief autobiographical recall and audiovisual clips was used to induce transient happy, neutral, and sad states. At the beginning of each block, participants were asked to remember an event in their lives that elicited one of the two target emotions, or a neutral state. Participants wrote down keywords that reminded them of that particular event, and were given 4 min to imagine themselves in that situation and to relive the emotions they felt at that time. To elicit happiness, participants were asked to think of an enjoyable moment with friends or children, or a time in which they cuddled or fooled around with a pet. To elicit sadness, participants were prompted to think of a person suffering or the death of a loved one. For the neutral block, participants had to recall a morning routine. The content of these memories was selected to match the theme of the 60-s clips they would watch immediately after.

Each clip included 11 pictures presented for 4.5 s, with a 1-s cross dissolve transitions in between. Images for the clips were taken from the International Affective Picture System (IAPS; Lang, Bradley, & Cuthbert, 2005), the Nencki Affective Picture System (NAPS; Marchewka, Żurawski, Jednoróg, & Grabowska, 2013; Riegel et al., 2016) or collected by the experimenters. All emotional pictures had been previously rated by 22 participants in a pilot study as respectively evoking sadness (*M* = 4.03, SE = 0.53) and happiness (*M* = 8.50, SE = 0.31) on a 11-point scale (1 = sad, 11 = happy). Examples of sad-inducing pictures are crying children or extreme poverty scenes, while happy-inducing images included smiling children, baby animals, or people dancing. Images used in the neutral clip depicted daily life objects such as tableware, and were selected from the IAPS and NAPS databases (valence ratings between 4.50 and 5.50; both databases used a 9-point scale). The presentation of pictures was accompanied by audio matching in valence. An excerpt from American Honey by Sam & Chesney was used for the happy clip; Adagio for Strings by Samuel Barber was used for the sad clips; and kitchen sounds were used for the neutral clip.

##### Emotion perception task

**Fig. 1:**
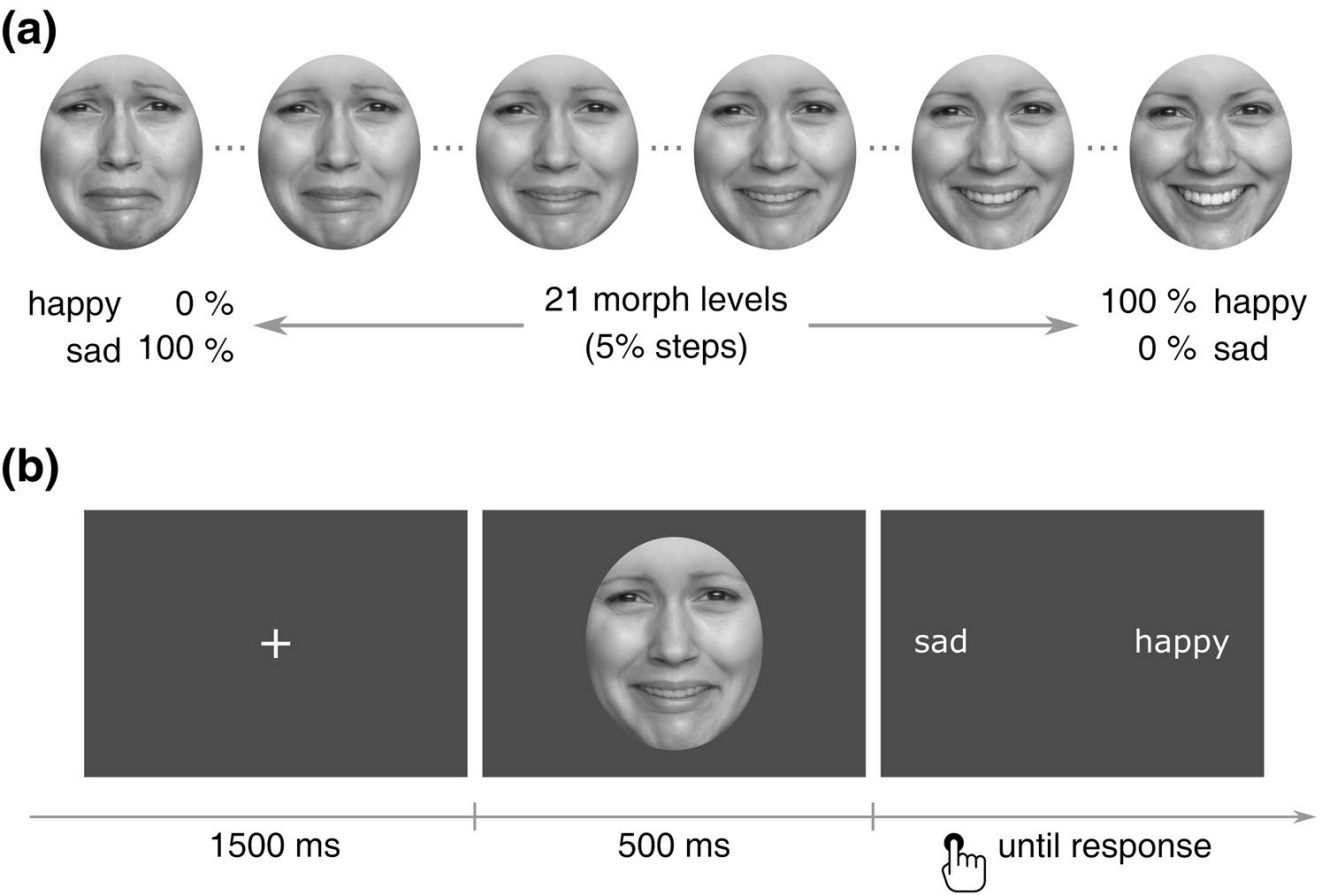
**a** Example of the continuum of sad-happy face morphs used in the emotion perception task. Due to copyright restrictions, the depicted face identity does not correspond to the one used in the study. **b** Example of a trial of the emotion perception task. The morph level presented was selected on a trial-by-trial basis using a 1-up/1-down adaptive procedure

A forced-choice psychophysical procedure was used to assess the perception of ambiguous emotional faces after each emotion induction. Twenty-one morphs of a female face were generated by mixing a happy facial expression with a sad facial expression in steps of 5%. The original stimuli were selected from the FACES database (Ebner, Riediger, & Lindenberger, 2010). Face morphs (496 × 659 pixels) were gray-scaled and embedded within a gray oval that occluded the hair and clothing (Fig. 1a).

Each trial of the task started with a fixation cross shown for 1500 ms, followed by the presentation of a face morph for 500 ms. Next, the response options ("happy", "sad") appeared on the right and left side of screen, respectively, until the participant made their response via key press (Fig. 1b). A 1-up/1-down adaptive method (Leek, 2001) was used to estimate the point on the happy-sad morph continuum at which observers were equally likely to judge the target emotion as happy or sad (point of subjective equality; PSE). PSEs have been used in previous studies to measure biases in emotion perception (Harris, Hayes-Skelton, & Ciaramitaro, 2016; Marneweck, Loftus, & Hammond, 2013). Here, lower PSEs are indicative of a higher tendency to perceive faces as happy (i.e. less happiness is required in the face to be equally likely judged as happy or sad). The adaptive procedure was implemented in MATLAB using the Palamedes Toolbox (Prins and Kingdom, 2018). One ascending and one descending interleaved staircases were set to select the morph stimulus on a trial-by-trial basis based on the participant’s previous response. Specifically, the morph level presented in each subsequent trial within a staircase was increased one step in the happy-sad morph continuum (i.e. 5% happier face) if the previous trial was a “sad” response, and decreased one step (i.e. 5% sadder face) if the previous trial was a “happy” response. The descending staircase started with 90–10% happy-sad morph, and the ascending with 30–70% happy-sad morph. The starting morph level for each staircase was determined based on pilot data, which showed that PSE with our stimuli was around the 60–40% happy-sad morph. Each staircase stopped after 8 reversals. A reversal is defined as a transition point within a staircase in which the participant switched their response from perceiving a happy face to a sad face, or vice versa. The PSE was estimated by averaging the morph levels of the last 5 reversals of both staircases. On average, participants completed 37.96 trials per block (SD = 5.83), which corresponds to a task length of approximately 1.5 min. A short task duration was important to ensure that PSEs were measured within the duration of the emotion induction effects.

### Procedure

The study followed a within-subject design. Prior to the experimental session, participants completed a series of online questionnaires to collect basic demographic information and to measure dispositional empathy and autistic traits. The scales Perspective-taking and Empathic Concern of the Interpersonal Reactivity Scale (IRI; Davis, 1980) were used to assess cognitive and affective empathy, respectively. In the German version of the IRI (Paulus, 2009), each scale consists of 4 items, scored on a 5-point Likert scale ranging from 1 (never) to 5 (always). The short German version of the Autism Spectrum Quotient (AQ; Baron-Cohen, Wheelwright, Skinner, Martin, & Clubley, 2001; Freitag et al., 2007) was used to assess individual differences in autistic traits. This version consists of 33 statements scored on a 4-point scale from "definitely agree" to "definitely disagree". All questionnaires were implemented in SoSci Survey (Leiner, 2018).

In the experimental session, participants completed three blocks of the emotional egocentricity paradigm. The three blocks corresponded to the happy, neutral and sad conditions, and each consisted of an emotion induction procedure, followed by the emotion perception task. Before and after each emotion induction, participants rated their current mood in a 9-point scale (− 4 = sad, 4 = happy). The order of happy and sad blocks was counterbalanced across participants, but neutral was always kept in the middle. Participants had a break of 5 min between blocks. Before the start of the first block, participants were introduced to the paradigm. To keep the aim of the experiment implicit, we presented a cover story, whereby each of the subsequent blocks contained two separate experiments, the first concerned with testing a new emotion induction procedure for future research, and the second with how we perceive emotions in a face. Participants also completed a shorter version of the emotion perception task to get familiar with the paradigm. The identity of the face morphs used in the practice was different from the face identity used in the main emotion perception task.

### Statistical analysis

The effectiveness of the emotion induction was checked with a 2 × 3 analysis of variance (ANOVA) on the participants' reported mood as dependent variable, and time (pre-induction, post-induction) and emotion condition (happy, neutral, sad) as within-subject factors. The Greenhouse–Geisser correction was used where applicable, and post-hoc t-tests with Bonferroni correction were performed to characterize the significant effects. In addition to the group analysis, mood ratings were individually screened to identify participants for which emotion induction was not successful. A total of 10 participants did not show the expected pattern of mood ratings, defined as: positive mood ratings after happy induction, negative mood ratings after sad induction, and mood ratings after neutral induction lying between those in the happy and sad conditions.

Two complementary approaches were used to examine the influence of affective state on emotion perception. First, a repeated-measures ANOVA with PSE as dependent variable and emotion condition (happy, neutral, and sad) as a within-subject factor was conducted. This approach reproduced the statistical analyses typically conducted in studies that use factorial designs to test the effects of induced mood on emotion perception (e.g., Lee, Ng, Tang, & Chan, 2008; Niedenthal, Brauer, Halberstadt, & Innes-Ker, 2000; Schmid and Schmid Mast, 2010). Given that this analysis takes emotion condition as a proxy of affective state, the data from the 10 participants whose mood ratings did not indicate successful emotion induction were excluded. This resulted in an analysis sample of 40 participants.

Arguably, the self-reported mood ratings are a more accurate indication of the participant's affective experience than the condition in which each PSE was measured. Therefore, stronger evidence of the influence of emotional state on emotion perception would be shown if post-induction mood ratings significantly predict PSEs regardless of emotion condition. To test this, we performed a linear mixed effects analysis of the relationship between mood ratings and PSEs. As fixed effects, we included the post-induction mood ratings as our main predictor of interest, and the pre-induction mood ratings as a control covariate. As random effects, we had intercepts for participants, as well as by-participant random slopes for the effect of post-induction mood ratings. P-values for the LMMs were computed based on Satterthwaite approximation for denominator degrees of freedom.

For this second analysis approach we did not exclude any participant based on the reported mood in each condition. However, one influential case was detected based on examination of *DFBETAS*, a standardized measure that indicates the level of influence single observations have on coefficient estimates (Nieuwenhuis, te Grotenhuis, & Pelzer, 2012). To obtain unbiased regression estimates, data from this participant was removed from the analysis sample and the linear mixed model was fitted a second time. Results reported below correspond to the model tested without this influential case (*n* = 49). Visual inspection of residual plots did not reveal any obvious deviations from homoscedasticity or normality.

The beta coefficients estimated for the random slope in the linear mixed model reflect the extent to which the self-reported mood influenced emotion perception for each participant. We used these coefficients as individual indices of emotional egocentricity, with more negative scores indicating stronger egocentric bias. To examine whether individual differences in empathy and autistic traits are related to the degree of self-projection during emotion perception, Pearson correlations were conducted between emotional egocentricity scores and the IRI and AQ scores.

Data and code to reproduce the statistical analyses are available at osf.io/5f4vn. All statistical analyses were run in R (R Core Team, 2016) and R studio (RStudio Team, 2019). We used the following R packages: *ez* for ANOVA (Lawrence, 2016); *lme4* (Bates, Mächler, Bolker, & Walker, 2015), *lmerTest* (Kuznetsova, Brockhoff, & Christensen, 2017) and *influence.ME* (Nieuwenhuis et al., 2012) for linear mixed effects analysis; *Hmisc* (Harrell and Dupont, 2018) for correlations; and *ggplot2* (Wickham, 2009) for figures.

## Results

### Manipulation check

**Fig. 2:**
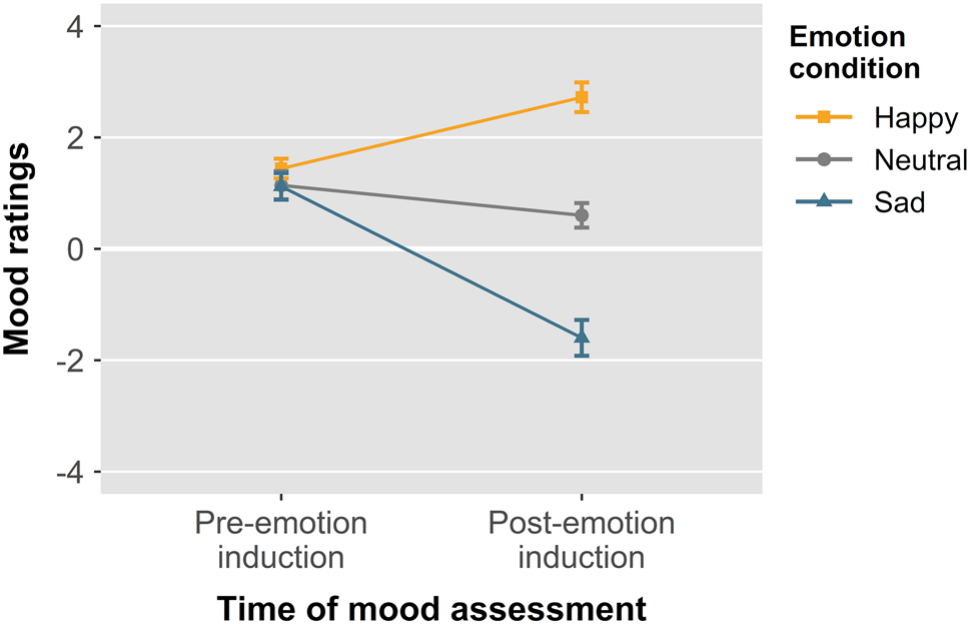
Mean self-reported mood ratings before and after each emotion induction procedure. Positive ratings represent happy state; negative ratings indicate sad state. Error bars represent within-subject 95% confidence intervals (Morey, 2008)

The repeated-measures ANOVA on mood ratings revealed a significant interaction between emotion condition and time, *F*(2, 98) = 126.32,* p* < 0.001, *η*_p_^2^ = 0.721. Planned pairwise comparisons confirmed that, while there were no significant differences in mood ratings before emotion induction (all *p*s > 0.16; pre-happy induction: *M* = 1.44, SE = 0.09; pre-neutral induction: *M* = 1.14, SE = 0.13; pre-sad induction: *M* = 1.12, SE = 0.12), participants felt significantly happier after happy induction (*M* = 2.72, SE = 0.13) than after neutral induction (*M* = 0.60, SE = 0.11), *t*(49) = 12.43,* p* < 0.001, 95% CI [1.78, 2.46], Cohen’s *d* = 1.76, and significantly less happy after sad induction (*M* = -1.60, *SE* = 0.16) compared to neutral induction, *t*(49) = − 12.66,* p* < 0.001, 95% CI [− 2.55, − 1.85], Cohen’s *d* = 1.79, as well as compared to happy induction, *t*(49) = 18.41,* p* < 0.001, 95% CI [3.85, 4.79], Cohen’s *d* = 2.60 (Fig. 2).

### Egocentric biases in emotion perception

**Fig. 3:**
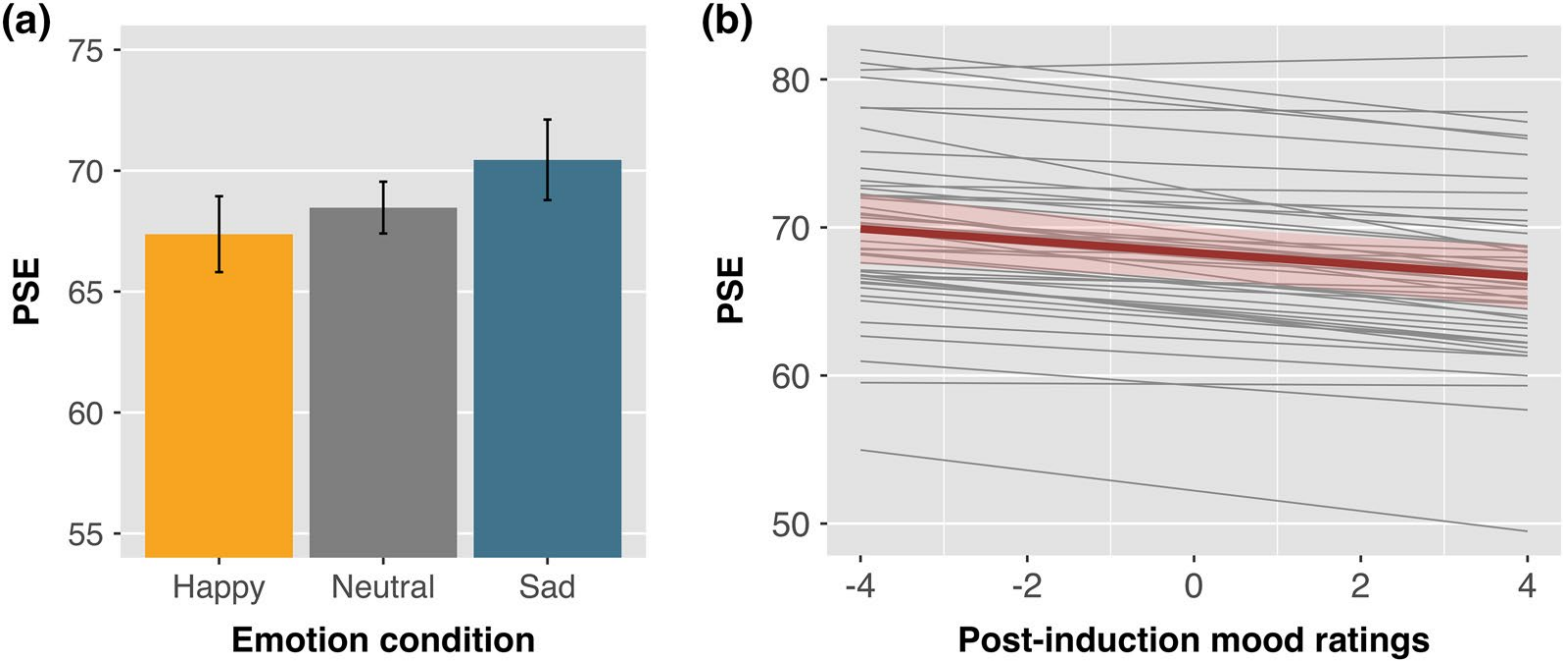
**a** Main effect of emotion condition on the point of subjective equality (PSE). PSEs indicate the percentage of happiness in the morph level at which participants were equally likely to judge the face as happy or sad. Lower PSEs are interpreted as a higher tendency to perceive happy expressions. Error bars represent within-subject 95% CI. **b** Effect of post-emotion induction mood on PSEs as estimated by the linear mixed model. The thick red regression line represents the predicted overall effect of mood (fixed effect), with 95% CI. A negative slope indicates that happier mood predicted higher tendency to perceive the faces as happy (i.e. lower PSEs). The estimated regressions lines for each individual participant (random effects) are represented with thinner gray lines

The ANOVA on the estimated PSEs showed a significant main effect of emotion condition, *F*(2, 78) = 4.67,* p* = 0.018, *η*_p_^2^ = 0.107, indicating that emotion perception was influenced by the participants' affective state (Fig. 3a). In line with the predicted mood-congruency bias, the PSE in the happy condition (*M* = 67.38, SE = 0.78) was descriptively lower than in the sad condition (*M* = 70.45, SE = 0.82), although this difference did not reach statistical significance after correction for multiple comparisons, *t*(39) = − 2.50,* p* = 0.051, 95% CI [− 5.57, − 0.58], Cohen’s *d* = 0.39. Differences in PSE between the neutral (*M* = 68.47, SE = 0.53) and sad conditions, and between the neutral and happy conditions, were also not statistically significant (all *p*s > 0.08). Though weaker, the main effect of emotion condition on PSEs remained significant even with inclusion of the full sample, *F*(2, 98) = 3.31,* p* = 0.049, *η*_p_^2^ = 0.063.

The influence of affective state on emotion perception was further demonstrated by the linear mixed model analysis, which showed that the mood participants reported after the emotion induction was a significant predictor of their PSE (estimate = − 0.40, SE = 0.18, *t* = − 2.23,* p* = 0.031, 95% CI [− 0.76, − 0.05]). As indicated by the negative slope, the happier participants were, the more likely they were to judge the emotional expressions as happy, indicated by lower PSEs (Fig. 3b). Pre-induction mood ratings did not significantly influence the PSEs (estimate = − 0.30, SE = 0.42, *t* = − 0.71,* p* = 0.48, 95% CI [− 1.12, − 0.53]).

### Gender differences

Given that both male and female participants made emotion judgments of a single female identity, we explored gender differences by re-running the main ANOVA with gender (2 levels: female, male) added as a between-subject factor. As before, there was a significant main effect of emotion condition, *F*(2, 76) = 4.85,* p* = 0.016, *η*_p_^2^ = 0.113, but neither the main effect of gender (*p* = 0.27), nor its interaction with emotion condition (*p* = 0.50), were statistically significant.

Adding gender and its interaction with post-induction mood ratings as predictors in linear mixed model analysis led to the same pattern of results: post-induction mood ratings remained a significant predictor of PSE (estimate = − 0.61, SE = 0.25, *t* = − 2.44,* p* = 0.019, 95% CI [− 1.09, − 0.12]), while gender (*p* = 0.13), its interaction with post-induction mood (*p* = 0.65), and the main effect of pre-induction mood ratings (*p* = 0.94) were not statistically significant.

### Associations between egocentric bias, empathy, and autism traits

**Fig. 4:**
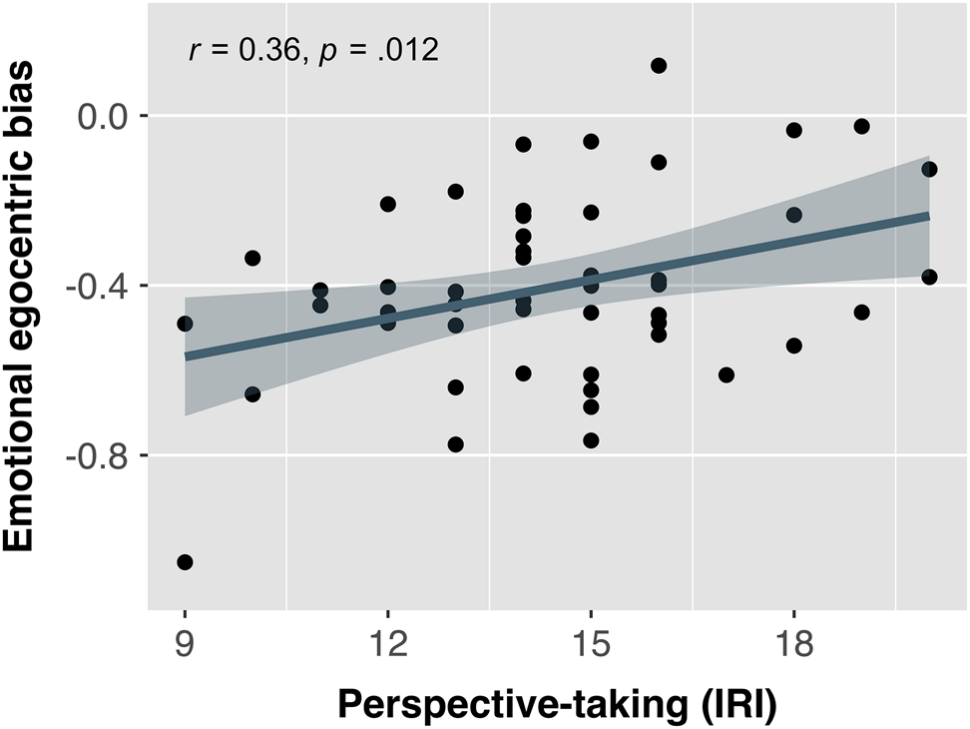
Correlation between emotional egocentricity and perspective-taking, as measured by the Interpersonal Reactivity Scale (IRI). Emotional egocentricity scores reflect the degree to which the own emotional state biased emotion perception, with more negative scores indicating stronger egocentricity. A positive correlation indicates that emotional egocentricity decreases with higher perspective-taking tendencies

The slopes estimated for each participant in the linear mixed effect model were used as index of emotional egocentricity, with more negative values indicating stronger egocentric bias. A weak but significant correlation was found between perspective-taking and emotional egocentricity bias, *r*(49) = 0.36,* p* = 0.012, indicating that higher perspective-taking abilities are associated with a reduced influence of the affective state on emotion perception (Fig. 4). Neither empathic concern nor autistic traits correlated significantly with emotional egocentricity scores (Table 1).

**Table 1:** Summary of correlations, means and standard deviations for the emotional egocentricity bias, IRI and AQ scores

Variable	*M*	SD	1	2	3
1. Emotional egocentricity bias	− 0.40	0.22			
2. Perspective-taking (IRI)	14.45	2.61	0.36* [0.08, 0.58]		
3. Empathic concern (IRI)	14.18	2.55	0.10 [− 0.19, 0.37]	0.39** [0.12, 0.61]	
4. Autistic traits (AQ)	8.61	4.80	0.01 [− 0.27, 0.29]	− 0.33* [− 0.56, − 0.05]	− 0.20 [− 0.46, 0.08]

*M* and SD represent mean and standard deviation, respectively. Values in square brackets indicate the 95% confidence interval for each correlation

*IRI* Interpersonal Reactivity Index, *AQ* Autism Spectrum Quotient

*Indicates *p* < 0.05

**Indicates *p* < 0.01

## Discussion

Research in social cognition has identified that one’s own emotional experiences are an important source of information to understand how another person is feeling. Previous studies detected egocentric biases when people make inferences about someone’s affective state based on information about the other’s context (Hoffmann et al., 2015; Silani et al., 2013; Steinbeis and Singer, 2014). The goal of the current study was to investigate whether emotional egocentricity also occurs when affective inferences rely on reading the person's emotional expressions.

Using a combination of brief emotion induction blocks with psychophysical measures of emotion perception, we were able to detect the occurrence of egocentric biases when participants judged ambiguous emotional faces. As hypothesized, facial expressions were more readily classified as happy when participants reported feeling happy as compared to sad. These results are indicative of a tendency to project the own affective states when making inferences about others’ emotions. Moreover, we found an association between perspective-taking and the extent to which the own mood influenced the emotion judgments, which provides evidence that these egocentric biases are related to social cognitive abilities.

Our results replicate the mood-congruent biases in emotion perception documented in the literature. One limitation of previous studies is that they could not disentangle whether the observed effects were due to the influence of the affective state experienced by the participant, or due to more general framing or priming effects, whereby exposure to affective stimuli may increase the readiness to process cues of the same valence. Evidence for perceptual biases provided in previous emotion induction studies consisted on comparing emotion recognition between groups exposed to induction of different affective states (Lawrie, Jackson, & Phillips, 2019; Lee et al., 2008; Niedenthal et al., 2000, Niedenthal, Brauer, Halberstadt, & Innes-Ker, 2001; Schmid and Schmid Mast, 2010). Although most studies included manipulation checks to demonstrate that groups differed in the experienced mood, statistical analyses could not rule out the possibility that the perceptual biases were caused by the mere exposure to an affective context, beyond whether or not this elicited an affective state to the participant. For example, Aguado, Martínez-García, Solís-Olce, Dieguez-Risco, & Hinojosa (2018) showed that perception of emotional expressions is enhanced when faces are introduced by a statement describing events that elicit affectively congruent emotions (e.g., angry faces are recognized faster when primed with the sentence "He notices someone has vandalized his car."). Arguably, this manipulation did not lead to significant changes in the participant's affective state, yet it elicited similar perceptual biases to those in mood induction studies.

Our study addressed this limitation by additionally using a statistical model that tested whether the participant's reported mood, instead of the emotion condition, predicted the PSEs. This statistical analysis allowed us to account for the inter-subject variability in mood ratings within conditions, which increased the power to detect any influences of affective state. The observed relationship between affective state and emotion perception (i.e., the happier the participant was, the more likely they perceived the faces as happy) provides a more direct support to the existence of mood-congruency effects, and strengthens the results from the ANOVA analyses, which despite of showing an overall effect of emotion condition on PSEs, post-hoc pairwise comparisons between conditions did not reach statistical significance.

The observation of egocentric biases extends previous literature on emotional egocentricity by showing that self-projection also occurs during perception-based emotion attribution. Relying on self-knowledge for understanding other's mental states can be an efficient heuristic, especially when only limited information about the other is available (Ready, Clark, Watson, & Westerhouse, 2000). Given that in our study participants had to make quick judgments about ambiguous emotional expressions, the own affect may have been used to guide their decisions. In fact, judgments of ambiguous faces, rather than clearer emotional expressions, have been shown to be more influenced by the participant's affective state (Cavanagh and Geisler, 2006). Future studies should assess to what extend individuals attribute their own affective states when making emotion judgments in more naturalistic situations in which additional contextual information and more time to correct for egocentric projections are available.

Importantly, the processes underlying egocentric biases in this study may be distinct to those previously observed in classic emotional egocentricity paradigms. In those tasks, participants are asked to make emotion judgments about a person based on information about the affective stimulation the other is exposed to, while at the same time receiving an affectively (in-)congruent stimulation themselves (e.g., Silani et al., 2013; von Mohr et al., 2019). To unbiasedly infer the other’s feelings, participants need to disengage from their own experience and adopt the other’s perspective, a process that relies on self-other distinction abilities (Lamm, Bukowski, & Silani, 2016; Steinbeis, 2016). Under those conditions, emotional egocentric biases have been interpreted as a failure to differentiate between the representations of one’s own affective states and those of the other (Hoffmann et al., 2016; Silani et al., 2013; Tomova et al., 2014).

In our paradigm, participants were not primed to think about their own affective state while judging the other’s emotional expressions, nor were asked to switch between self and other processing. In this context, the intrusion of the self-affect was more implicit and unconscious, and participants may have not actively tried to inhibit the influence of their own state nor tease apart the self- and other-representations as in previous emotional egocentricity tasks. Therefore, while our results support the idea that the own experience is recruited when reading others’ emotions, egocentric biases here should not be interpreted as an index of the participants’ self-other distinction abilities.

Instead, egocentric judgments during emotion reading could be related to the participants’ general disposition to shift attention towards the other’s experience during social interactions. Specifically, we found a small but significant correlation between emotional egocentricity and individual differences in the perspective-taking scale of the IRI, such that individuals with a higher predisposition to adopt the point of view of others were less influenced by their own affective states. This goes in line with results of a recent meta-analytic study that found a positive association between dispositional perspective-taking and emotion recognition accuracy (Israelashvili, Sauter, & Fischer, 2019). A higher tendency to engage in perspective-taking may lead to more attention deployed to the other during processes of mental state inference, thus minimizing the influence of the own affect and facilitating a more accurate representation of the other’s experience. Supporting this hypothesis, perspective-taking tendencies have been related to the extent to which participants focus on the other person’s perspective relative to their own in a visual perspective-taking task (Bukowski and Samson, 2017). Egocentric biases in our study may have thus partly resulted from a lower disposition to amplify the representation of the other, rather than a failure in inhibiting the self-representation.

While our results suggest that components of cognitive empathy are linked to the tendency to project one’s own emotions onto others, we did not find evidence for an involvement of affective empathy. Specifically, individual differences in empathic concern did not significantly correlate with the degree of emotional egocentricity. Though conclusions from null results should be drawn cautiously, our finding parallels previous studies in which emotional egocentricity was also not associated with empathic concern (Hoffmann et al., 2016) nor with other factors related to affect sharing such as alexithymia (von Mohr et al., 2019). Instead, emotional empathy seems to be more related to altercentric biases, that is, to the influence of the other’s emotions on the judgment of our own affective states (Hoffmann et al., 2016).

Finally, we did not find evidence for an association between emotional egocentricity and autistic traits. Even though stronger egocentric biases have been reported in ASC (Bradford et al., 2018; Pearson et al., 2013), as well as in individuals from the general population with high autistic traits (Brunyé et al., 2012), these were mainly detected in cognitive mentalizing tasks. The only study known to us that specifically investigated emotional egocentricity did not find differences in the magnitude of egocentric biases between individuals with ASC and controls (Hoffmann et al., 2016). Taken together, these findings point to a dissociation in the impact of autistic traits on cognitive and affective mentalizing: while people with high autistic traits may show difficulties when inferring others’ knowledge and beliefs, autistic traits do not seem to significantly impact the capacity to overcome self-projections during emotion inferences.

Some limitations of the study should be mentioned. First, only face morphs of one female identity were used in the emotion perception task. Previous research has shown that factors such as liking or the perceived similarity with the other influence the degree to which people project their own mental states (Davis, 2017; O’Brien and Ellsworth, 2012). As such, one could expect that female participants in our study would have shown stronger egocentric biases than male participants as they were rating same-gender targets. Our exploratory analysis did not show significant gender differences in the influence of affective state on emotion judgments. Nevertheless, given our sample size, this result should be interpreted with caution, as we had limited power to test the moderation by gender. Moreover, due to our task design, we could not have distinguished whether potential gender effects would reflect in-group biases, different motivational attitudes towards the target, or general gender differences in emotion attribution. By including more diversity of face identities and controlling for the perceived similarity with the target, future studies should address this issue and improve generalizability of the findings.

Second, participants made binary emotion choices in the emotion perception task, while some of the morphs may have actually been perceived neither as happy nor sad. The 2-alternative forced-choice design was chosen to increase the likelihood to detect egocentricity effects and limit the task duration. To increase ecological validity, new emotional egocentricity paradigms could offer a wider range of emotion options. In addition, future task designs would benefit from adding a control condition in which participants make non-emotion judgments about the target while being under the emotion induction effects. This would allow to draw more definite conclusions about the specificity of the mood effects in relation to emotion reading vs. other mood-congruent perceptual biases unrelated to social cognitive processes.

Finally, although on average the emotion induction procedure worked, there was variability in the effectiveness of the manipulation, with some participants not reporting the expected mood in each condition. Moreover, even though the assessment of PSEs was completed within a short period of time (approx. 1.5 min) right after each emotion induction, we cannot rule out that the strength of the induced affect decreased throughout the task. Attenuated affective self-representations may have reduced the chance of observing a bias. In future work, the use personalized and longer-lasting forms of emotion induction could facilitate the detection of mood-congruency effects.

Notwithstanding these limitations, our study implemented a novel approach to quantify egocentric biases during emotion attribution. The adaptive psychophysical task allowed us to detect subtle changes in the tendency to perceive emotional faces as happy when participants were in different affective states. Unlike some of the previous mood-congruence studies (e.g., Harris et al., 2016; Lee et al., 2008; Niedenthal et al., 2000, 2001; Schmid and Schmid Mast, 2010), we used a within-subject design, which gave us the possibility to estimate individual bias scores and explore associations with socioemotional traits. The simplicity of the paradigm makes it appropriate to be used in emotional egocentricity research with clinical samples.

In conclusion, the current study established the existence of egocentric biases when reading facial expressions of emotion. We showed that individuals are more likely to perceive ambiguous happy-sad face morphs as happy when they are feeling happy as compared to when they are sad. More importantly, the magnitude of the egocentric bias was associated with perspective-taking tendencies, which suggests that socio-cognitive processes may underlie mood-congruency biases in emotion perception. Our study extends the literature on emotional egocentricity by showing that self-projection also occurs when we rely on the other’s emotional expressions for understanding their affective state.

## Data Availability

The datasets generated during and/or analyzed during the current study are available in the Open Science Framework repository: osf.io/5f4vn.
